# Enhanced Recognition Memory after Incidental Encoding in Children with Developmental Dyslexia

**DOI:** 10.1371/journal.pone.0063998

**Published:** 2013-05-23

**Authors:** Martina Hedenius, Michael T. Ullman, Per Alm, Margareta Jennische, Jonas Persson

**Affiliations:** 1 Department of Neuroscience, Uppsala University, Uppsala, Sweden; 2 Brain and Language Lab, Department of Neuroscience, Georgetown University, Washington, D.C., United States of America; 3 Aging Research Center, Karolinska Institute and Stockholm University, Stockholm, Sweden; University of California, San Francisco, United States of America

## Abstract

Developmental dyslexia (DD) has previously been associated with a number of cognitive deficits. Little attention has been directed to cognitive functions that remain intact in the disorder, though the investigation and identification of such strengths might be useful for developing new, and improving current, therapeutical interventions. In this study, an old/new recognition memory paradigm was used to examine previously untested aspects of declarative memory in children with DD and typically developing control children. The DD group was not only not impaired at the task, but actually showed superior recognition memory, as compared to the control children. These findings complement previous reports of enhanced cognition in other domains (e.g., visuo-spatial processing) in DD. Possible underlying mechanisms for the observed DD advantage in declarative memory, and the possibility of compensation by this system for reading deficits in dyslexia, are discussed.

## Introduction

Developmental dyslexia (DD) is characterized by unexpected difficulties with reading, in the context of typical educational opportunities and intact intellectual and sensory abilities [1]. The disorder, which has a strong genetic component [2], has been estimated to affect about 5–10% of children [3]. Children with DD have difficulties with written word recognition and phonological decoding (using letter-sound mapping knowledge to decode novel words), which may result from underlying phonological impairments [4], [5]. In addition, DD has been associated with a number of other deficits [6], [7], including of working memory [8], [9], executive functions [10], motor function [11], implicit sequence learning [12], [13], [14], and artificial grammar learning [15] as well as problems with other aspects of language that appear to be primary in nature (i.e. not only a consequence of impaired reading) [16], [17], [18].

Although much attention has been given to cognitive deficits associated with DD, it is evidently not the case that all cognitive functions are impaired in the disorder. Knowledge about what is well-functioning may be as important for increasing our understanding of the disorder as knowledge about what is impaired. The investigation and identification of cognitive strengths associated with DD could be used for developing new, and improving current, therapeutical interventions. In this study, we examine a previously untested aspect of declarative memory in children with DD, namely recognition memory after incidental encoding.

Declarative memory encompasses memory for factual knowledge (semantic memory) and personally experienced events (episodic memory) [19], [20]. It relies on a network of brain structures in which the medial temporal lobe, including the hippocampus and the nearby cortices, plays a critical role [21], [22]. Other brain structures of importance in this system include portions of frontal cortex, which play a crucial role in encoding and recall in declarative memory [23], [24]. Depending on the specific paradigm used to assess declarative memory, the relative demand on executive functions underlying encoding strategies and recall of information may be increased or decreased. For example, increased demands on working memory and executive functions have been associated with intentional as compared to incidental encoding, and with free recall as compared to recognition [25].

Few studies have directly tested declarative memory functions in DD, and those that have done so have yielded inconsistent findings. Whereas some studies have reported that declarative memory is normal in the disorder [26], [27] others have found a deficit [28], [29], [30]. Consistent with the well-documented phonological impairments associated with DD, some of the contradictory findings appear to be explained by whether verbal or non-verbal material is used to test declarative memory. For example, learning and retention of non-verbal information has been shown to be intact in the paired-associate learning task [31], [32], which is a classic declarative (episodic) memory paradigm. The same studies reported impairments at learning when verbal stimuli were used [31]. However, these group differences disappeared when phonological impairments were controlled for, suggesting that the impairment might not be related to declarative memory per se but to underlying phonological problems. Accordingly, in a third study, paired-associate learning with verbal stimuli was found to be intact in DD when short, high-frequency words (which minimizes the effect of phonological processing problems), were used [33].

Another factor that appears to affect the performance of dyslexic participants in tasks probing declarative memory is the extent to which test performance depends on the use of intentional encoding strategies. In line with previous evidence suggesting problems with executive functions in DD [10], one study found that the declarative memory impairment in the DD group appeared to be explained by an inability to develop efficient strategies for encoding [29]. In addition, free recall of previously presented material may be particularly challenging for individuals with DD [9], [34].

Declarative memory is a complex phenomenon that depends on the integrity of several underlying functions. Accordingly, a research program aimed at elucidating the status of declarative memory in DD may benefit from a systematic investigation of these underlying functions. One aspect of declarative memory that still remains untested in DD is recognition memory after incidental encoding. Crucially, in this paradigm, subjects are unaware of the subsequent memory test, and incidental encoding is promoted by, for example, a semantic categorization task. Thus, potential group differences in intentional encoding strategies should not be a confounding factor in this task. Moreover, this paradigm does not rely on free recall of information but rather on the identification of a specific item as “old/seen before” or “new/not seen before”. These characteristics may make this paradigm particularly suitable for revealing aspects of declarative memory that might function well in DD. Despite these advantages, this paradigm has not yet been used in DD. The present study was designed to fill this gap in the literature.

## Methods

### Ethics Statement

The study was approved by the ethical review board in the city of Uppsala. All parents or guardians provided informed written consent; children provided informed written assent and received a cinema ticket for their participation.

### Participants

Twelve children with developmental dyslexia (DD) and 17 typically developing (TD) control children participated in the study. The two groups did not differ in sex, age and handedness (see Table 1 for participant characteristics of the final set of participants included in statistical analyses).

**Table 1:** Participant demographics and cognitive characteristics.

Demographics	DD	TD	*t*	*p*
N	11	17	–	–
Age in years	11.0 (0.71)	11.0 (0.49)	0.28	.78
Sex (f/m)	5/6	5/12	*χ^2^ = *0.75	.39
Handedness	85.1 (16.4)	92.2 (10.2)	1.4	.17
Cognitive scores				
PIQ	87.3 (12.5)	97.1 (15.0)	1.8	.085
Phonological decoding	1.82 (0.87)	5.24 (1.15)	8.4	<.0001
Orthographic reading	2.0 (1.18)	5.76 (1.15)	8.4	<.0001
Nonword repetition	106 (5.3)	111 (5.4)	2.4	.026
TROG	17.9 (1.20)	18.9 (0.70)	2.7	.012
PPVT	150 (15.9)	160 (13.5)	1.8	.089

DD = Children with developmental dyslexia, TD = Typically developing control children, PIQ = Performance IQ; TROG = Test for reception of grammar; PPVT = Peabody picture vocabulary test. Standard deviations are shown in parentheses.

Children with DD were recruited via speech-language pathology clinics in the cities of Stockholm, Uppsala, Gävle and Västerås, in Sweden. All children with DD had been independently tested and diagnosed with dyslexia by a certified speech-language pathologist within 1.5 years prior to participation in the study. The TD group consisted of a subset of children who were recruited from schools in and around the cities of Stockholm and Uppsala as part of a larger study on memory and language in typically developing children. All children in the study were reported by their parents to be monolingual Swedish-speaking, to have normal (or corrected to normal) vision and hearing, and to have no known cognitive or motor impairment, apart from reading problems in the DD group.

In order to confirm reading problems in the DD group, and the lack thereof in the TD group, two reading tests, assessing phonological decoding and orthographic reading, respectively, were administered on the same day as the declarative memory task. These tests were paper and pencil Swedish adaptations [35] of the computerized phonological decoding and orthographic reading tasks used by Olson, Forsberg, Wise, & Rack [36]. In the phonological decoding test, the task was to decide, and underline with a pencil, which one of three or four pseudo-words was a pseudo-homophone of a real word. (i.e. “sounds” like a real word). The score was the number of correctly identified pseudohomophones within two minutes, with a maximum score of 80. In the orthographic reading test, participants were asked to underline the true word in true word-pseudohomophone pairs. Because the phonological codes for the pairs were identical, the word and its pseudohomophone would be pronounced the same in Swedish. Thus, in order to make a correct response subjects had to use word-specific orthographic knowledge. The score was the number of correctly chosen words in two minutes, with a maximum score of 120.

All TD children had stanine scores ≥4 out of 9 on both reading tests (corresponding to performance at or above –0.75 *SD*). All children in the DD group had stanine scores of ≤3 on both tests, except for one child who had a stanine score of 5 on the orthographic reading test. Because previous evidence suggests that the phonological decoding problems characteristic of DD can sometimes occur together with intact or even superior orthographic skills [37], this child was still included in the DD group.

In addition to these reading tests, all children were also tested on nonword repetition [38], understanding of grammatical structures [39], [40], and receptive vocabulary [41] (see Table 1). The DD group performed significantly worse than the TD group on both the nonword repetition test and the grammar test. Receptive vocabulary, by contrast, did not differ significantly between groups (Table 1).

Performance IQ (PIQ), which was assessed by Raven’s Standard Progressive Matrices Plus [42], was included for descriptive rather than exclusionary purposes in this study, since we wanted our DD sample to reflect the population of children who are clinically identified and diagnosed with DD in Sweden. In line with evidence suggesting that there is only a weak, if any, relationship between PIQ and the reading problems characteristic of dyslexia [43], [44], PIQ is not used as an exclusionary factor for a clinical diagnosis of DD in Sweden. The PIQ range in the DD group was 70–115 and the TD range was 80–140. Because the group difference in PIQ approached significance (*p* = .085; Table 1), PIQ was controlled for in all statistical analyses.

One child in the DD group was excluded from statistical analyses because he was an outlier on the recognition memory test, with a mean d’ score of 0.19, which was equal to − 3 *SD* from the mean of all children. All other children fell within 2 *SD*s of the mean. Thus, the final set of participants on which analyses were performed consisted of 11 children in the DD group and 17 children in the TD group.

### Stimuli and Procedure

Declarative memory was tested with an object recognition memory task developed by the Brain and Language Lab at Georgetown University. Similar tasks have previously been shown to engage the network of brain structures underlying declarative memory, including medial temporal lobe and frontal structures, for both non-verbal and verbal stimuli [45], [46].

**Figure 1:**
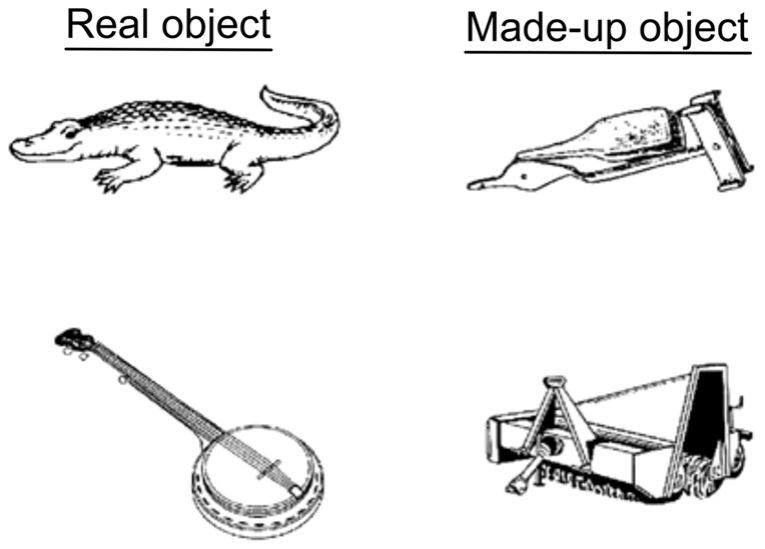
Examples of the real and made-up objects used as stimulus materials.

The object recognition task consists of three phases; i) incidental encoding, ii) recognition 10 minutes after encoding and iii) recognition 24 hours after encoding. The stimuli were black-and-white line drawings of real objects and made-up objects (Figure 1). The images used for both the real and made-up objects were taken from a variety of sources. For the real objects, items were drawn from, and modified as necessary, various clipart galleries (including free websites and purchased collections), and from a previous study by Snodgrass et al. [47]. For the made-up objects, items were selected and modified from previous studies by Eals and Silverman [48], Laine et al. [49] and Williams and Tarr [50]. The images of made-up objects were selected based on their nameability (that is, low nameability) determined through previous pilot work. All images were resized, touched up, rotated, and/or converted to black-and-white to create the final set of stimuli. The items were presented in a pseudo-randomized order, with no more than 3 consecutive real or made-up objects.

There were three different sets of objects used in this task: i) those presented in the encoding phase (as well as in subsequent phases), ii) those used as foils in the 10 minute recognition phase and iii) those used as foils in the 24 hour recognition phase. Each of these three sets of objects consisted of 32 real objects and 32 made-up objects.

Testing took place in a quiet room under normal classroom lighting conditions. The stimuli were presented on an LCD screen of a portable PC computer running Windows, using E-Prime version 1.2. (Psychology Software Tools). The physical size of the images was 13.7×10.3 centimeters, and the viewing distance was approximately 50 centimeters. Participants were instructed to place their left and right index fingers on the designated buttons on a serial response box (E-prime SRBox) that was placed in front of them, and to make a response by pressing one of these buttons. Preceding each stimulus, a crosshair appeared in the center of the screen for 1000 milliseconds (ms), followed by the item for 500 ms, also in the center of the screen. In cases where the participant responded before 500 ms, the item remained onscreen until the 500 ms finished, to equalize presentation duration across stimuli and subjects. After the item presentation, the crosshair reappeared on the screen until the subject responded, or up to 4500 ms. As soon as the subject made a response through the SRBox, a 200 ms advance tone sounded, followed by 800 ms of fixation. If instead the subject made no response within the 4500 ms response period, a 400 ms time-out tone sounded, followed by 600 ms of fixation. After this the 200 ms advance tone sounded followed by 800 ms of fixation. The next item then began with 1000 ms of fixation.

In the incidental *encoding* phase, participants were told they were going to be presented with pictures of “real” and “made-up” objects on the screen. They were asked to indicate, through a button press, whether the object was real (existed in reality) or made-up (did not exist in reality). Similar categorization tasks have previously been used to promote incidental word encoding [51]. The instructions included 2 sample items to ensure that all participants understood the task and correctly mapped the response buttons. The instructions and sample items were followed by 3 practice items with the same timing parameters as the test items. All instructions were visible on the screen and were simultaneously read aloud to the participants. A reminder appeared at the bottom of the screen throughout the task indicating the mapping of the SRBox buttons (i.e. “real”/”made-up”).

The incidental encoding phase was followed by a 10 minute break during which subjects were encouraged to stretch their legs or have a snack. Just before the subsequent *10 minute recognition* phase, subjects were told they were going to see pictures of real and made-up objects again, some of which they saw previously and some of which they did not. They were asked to indicate, through a button press, whether or not they had seen the object earlier. As in the encoding phase, the instructions included sample items, which were followed by practice items. Presentation and timing was the same as in the encoding phase, but the reminder “real”/”made-up” was changed to “yes”/“no”. Additionally, the question “seen before?” was always displayed on the screen, above the “yes” and “no” options. Finally, after a 24 hour interval (+/−1 hour) subjects were given the *24 hour recognition* phase of the task. Instructions, presentation and timing were the same as in the 10 minute recognition phase.

Responses were captured using E-prime version 1.2. Two versions (A and B) of the task, for which the response buttons for “real”/“made-up” and “yes”/“no” were reversed, were assigned to consecutive participants in each group. Testing took place in a quiet room in the child’s school or home over the two consecutive days. Each test session took about 70–90 minutes, including the administration of reading, language and performance IQ tests.

Reaction times (RTs) were calculated for correct responses only, and filtered by excluding responses faster than 300 milliseconds (ms) or greater than 4500 ms. Median RTs were used in order to avoid undue influence from outlier RTs. D-prime (d’ = *z* hits – *z* false alarms), which takes response bias into account, was used to assess recognition memory accuracy. We report unadjusted *means* and *SD*s unless otherwise indicated. Partial eta-squared (η_ρ_^2^) is used as the measure of effect size where appropriate.

## Results

### Categorization Accuracy and RT during the Incidental Encoding Task

Potential group differences (DD vs. TD) in the incidental encoding task were tested with one-way ANCOVAs (with PIQ as a covariate), with either categorization accuracy (percent correct) for real vs. made-up, or reaction times of correct responses (RTs), as the dependent variable. The DD and TD groups did not differ significantly in accuracy, though the DD group showed somewhat better performance, a pattern that approached significance (DD *mean* = 86.4%, *SD* = 13.9%; TD *mean* = 73.9%, *SD* = 20.3%, *F*(1,25) = 3.442, *p* = .075, η_ρ_^2^ = .121). The two groups did not differ in their reaction times (DD *mean* = 958 ms, *SD* = 150 ms; TD *mean* = 862 ms, *SD* = 241 ms, *F* <1). Speed-accuracy tradeoff effects during encoding were examined with a partial correlation analysis across subjects (controlling for PIQ) of RT against accuracy. This analysis revealed no association between response speed and accuracy, either across all children (*r*(25) = −.163; *p* = .417) or within any of the two groups (DD group: *r*(8) = −.274, *p* = .444; TD group: *r*(14) = −.233, *p* = .384).

### Recognition Memory Accuracy and RT

Potential group differences in recognition memory accuracy across the two recognition sessions (10 minutes and 24 hours) were examined with a 2 (group: DD vs TD)×2 (session: 10 minutes vs 24 hours) ANCOVA with d’ scores as the dependent variable. Because the DD group’s trend of better performance at distinguishing real and made-up objects in the incidental encoding task could potentially confound performance at recognition, semantic categorization performance during encoding was included along with PIQ as a covariate.

**Figure 2:**
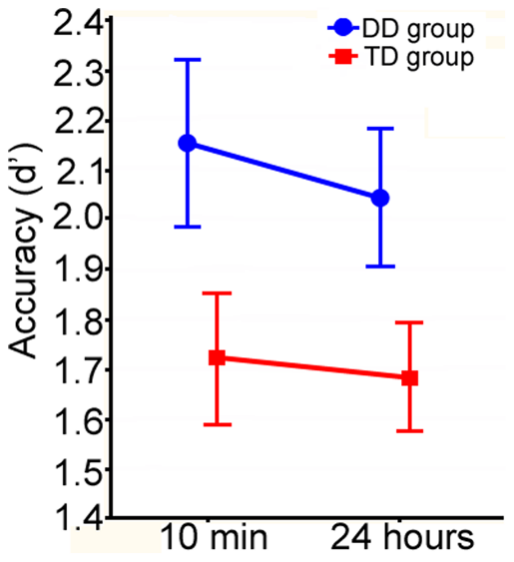
Adjusted means and standard errors for children with developmental dyslexia (DD) and typically developing control children (TD).

Analyses revealed that the DD group had better recognition memory, as compared to the TD group, across both recognition sessions (Figure 2): the 2×2 ANCOVA produced a significant main effect of group (*F*(1,24) = 4.45, *p* = .045, η_ρ_^2^ = .156), with no main effect of session (*F* <1), and no group by session interaction (*F* <1).

Reaction times, by contrast, did not differ significantly between the two groups (mean RTs across both recognition sessions: DD *mean* = 1004 ms, *SD* = 124 ms; TD *mean* = 923 ms, *SD* = 168 ms). The 2 (group: DD vs TD)×2 (session: 10 minutes vs 24 hours) ANCOVA with RT as dependent variable (controlling for PIQ and encoding accuracy) produced no significant effects (main effect of group: *F*(1,24) = 1.41, *p* = .248, η_ρ_^2^ = .055; main effect of session: *F* <1; group×session interaction: *F* <1).

### Control Analyses

In order to rule out alternative explanations to the observed DD recognition memory advantage, a set of control analyses was performed, with the aim of investigating potentially confounding factors.

First, we asked if group differences in speed-accuracy tradeoff during the recognition memory task could explain the present findings. This question was addressed by partial correlation analyses (controlling for PIQ and encoding accuracy) of RT against d’ scores within each recognition session separately. These analyses revealed no evidence of speed-accuracy tradeoffs in either of the two sessions, across all children or within either of the two groups (10 minute recognition session: all children: *r*(24) = .017, *p* = .923; DD group: *r*(7) = −.358, *p* = .309; TD group: *r*(13) = .005, *p* = .984; 24 hour recognition session all: *r*(24) = −.170; *p* = .395; DD group: *r*(7) = −.396, *p* = .257; TD group: *r*(13) = −.200, *p* = .457).

Second, because the two groups were somewhat unbalanced with respect to sex (Table 1), it is possible that the group difference in recognition memory was an artifact produced by the greater number of boys in the TD group. We therefore added sex as a covariate to the model. This 2×2 ANCOVA with three covariates (semantic categorization accuracy, PIQ, and sex) yielded the same pattern as that reported above: a main effect of group, with the DD group showing better recognition memory than the TD group (*F*(1,23) = 4.30, *p* = .049, η_ρ_^2^ = .158). No other main effects or interactions were significant (main effect of sex: *F*(1,23) = 1.68, *p* = .207, η_ρ_^2^ = .068, main effect of session: *F* <1, group by session interaction: *F* <1).

Third, the effects of PIQ were more carefully examined. First, we performed a correlation analysis between PIQ and semantic categorization performance (which was the second covariate in the main ANCOVA). This analysis revealed that there was no correlation between PIQ and semantic categorization, across groups or within groups (all *p*s >.6). Next, the relationship between PIQ and the dependent variable (recognition memory) was investigated. There was no correlation between recognition memory mean d’ scores and PIQ, either across all children or within the TD group (*p*s >.6). However, there was a marginally significant association between PIQ and recognition memory performance in the DD group (*r* = .594, *p* = .054), confirming the desirability to control for PIQ in the group comparison of recognition memory.

Finally, we followed up on these analyses by creating new DD and TD groups that were matched one-to-one on PIQ. These groups were created by including all DD children for which there was a TD child with an identical PIQ standard score. If there were more than one TD option for a particular DD child (this was the case for one DD child in our data set), we selected the one that would also balance the groups with respect to sex. There were 5 children (3 boys and 2 girls) in each group. All DD children had mean stanine reading scores ≤2 and all TD children had mean stanine reading scores ≥4. The PIQ range was 85–115. These groups were then compared with Mann-Whitney U tests on recognition memory d’ scores and reaction times. The results confirmed the pattern observed in the main analysis with a significant DD advantage at recognition memory accuracy (main effect of group across both sessions: *p* = .022) and no group difference in RT (main effect of group across both sessions: *p* = .676).

### Correlations between Reading Performance and Recognition Memory

Next, the relationships between reading performance and recognition memory within the two groups were examined. We performed partial correlation analyses (controlling for PIQ and encoding accuracy) between the mean d’ score across both sessions and the combined standardized raw scores from the phonological decoding and orthographic reading tests. These analyses revealed a marginally significant association between recognition memory and reading performance in the DD group *(r*(7) = .644, *p* = .061), whereas there was no such association in the TD group (*r*(13) = −.081, *p* = .774). The difference between the correlations between recognition memory and reading in the two groups approached significance (*p* = .056).

### Analyses of Object-type Effects

Finally, to examine whether the two groups differed with respect to potential effects of object-type, we performed 2 (object-type: real vs made-up)×2 (group: DD vs TD) ANCOVAs (controlling for PIQ and encoding accuracy) for each of the two recognition sessions. The ANCOVA for the 10 minute session produced a marginally significant main effect of group (*F*(1,24) = 4.10, *p* = .054, η_ρ_^2^ = .146), with better performance for the DD group compared to the TD group (real objects: DD d’ *mean* = 3.18, *SD* = 0.72; TD d’ *mean* = 2.71, *SD* = 0.81; made-up objects: DD d’ *mean* = 1.48, *SD* = 0.60; TD d’ *mean* = 1.15, *SD* = 0.62), a significant main effect of object-type (*F*(1,24) = 6.39, *p* = .018, η_ρ_^2^ = .210), with superior memory for real compared to made-up objects, and no object-type×group interaction (*F* <1). Thus, in the 10 minute session, the DD group showed somewhat better performance for both object types, and both groups showed better performance for real compared to made-up objects.

In the 24 hour session, there was again a marginally significant main effect of group (*F*(1,24) = 4.0, *p* = .057, η_ρ_^2^ = .143), with better DD compared to TD performance (real objects : DD d’ *mean* = 2.06, *SD* = 0.31; TD d’ *mean* = 2.02, *SD* = 0.26; made-up objects: DD *mean* = 1.95, *SD* = 0.85; TD *mean* = 1.67, *SD* = 1.49), but no effect of object-type (*F*(1,24) = 1.33, *p* = .261, η_ρ_^2^ = .052). The object-type×group interaction was marginally significant (*F*(1,24) = 3.99, *p* = .057, η_ρ_^2^ = .142). Although the lack of significance for this interaction does not support follow-up analyses, the *mean* values suggest that the DD compared to TD advantage in the 24 hour session may be larger for the made-up objects than the real objects.

## Discussion

The aim of the present study was to investigate a previously untested aspect of declarative memory in children with DD, namely recognition memory after incidental encoding. Based on previous evidence indicating that declarative memory impairments in DD may be related to less efficient encoding strategies [29] and/or problems with free recall [9], [34], we predicted that the present paradigm would yield intact performance in the DD group. Recognition memory was found to be not only intact, but even superior, in the DD group as compared to the TD group. These results were not driven by group differences in PIQ, sex or speed-accuracy trade-off effects.

Developmental dyslexia has previously been associated with a number of linguistic and non-linguistic deficits (see introduction). The present findings indicate that this condition may also entail an enhancement of certain cognitive functions. These findings complement previous reports suggesting enhancements in other aspects of cognition in DD, namely visuo-spatial processing [52], [53]. Although the mechanisms underlying the observed enhanced performance at recognition memory are still unknown, we consider several possible explanations.

One explanation that has been proposed to account for enhanced visuo-spatial processing in DD [52], and that could plausibly play a role in the findings here, is that the superior recognition memory performance of the DD group was an effect of an enhanced ability to create semantic labels or associations for the “non-nameable” made-up objects. According to this account, a frequent use of semantic substitutions to compensate for lexical retrieval deficits may make the DD group more adept at creating semantic labels or associations for objects that are difficult to describe, which in turn might have a positive impact on memory performance. This hypothesis would predict performance in the DD group to be enhanced, compared to the TD group, specifically for the made-up objects. Indeed, the marginally significant group×object-type interaction in the 24 hour recognition session hints at the possibility of a group difference in the consolidation of made-up objects (with somewhat better performance in the DD group). However, this hypothesis seems somewhat inconsistent with the fact that the two groups showed an equally large advantage for real as compared to made-up objects in the 10 minute session. Future studies including larger samples might help clarify the nature of these object-type effects and how they may relate to dyslexia.

Alternatively, it may be the case that the superior performance in the DD group was not a result of an enhancement in this group, but rather the result of relatively impaired performance in the TD group. According to the neuronal recycling hypothesis [54], learning to read entails a tradeoff in which the building up of a sight-word lexicon takes place at the cost of certain other visual skills. Support for this hypothesis comes from the tradeoff between reading and other aspects of visual cognition that has been observed in illiterate adults who learn to read [55] and in dyslexic participants following remediation [56]. This hypothesis would predict that the DD advantage at recognition memory observed in the present study would diminish as the dyslexic children improve their reading ability. Although the design of the present study does not allow us to further investigate this hypothesis, a future follow-up study may be warranted to specifically test this prediction.

A third possible explanation for the present findings is that they reflect intricate interactions between the procedural and declarative memory systems. Converging evidence from human and animal studies suggest that the procedural and declarative memory systems rely on at least partly dissociable neural substrates and support different cognitive functions [57]. However, the two systems do not work in isolation. Rather, the relative involvement of the procedural and declarative systems during cognitive task performance appears to be modulated by the to-be-learned material on the one hand [6], [58], [59], [60], and by complex “competitive” and “cooperative” interactions between the two systems on the other (for reviews see [6], [61], [62]). Competitive interactions, sometimes referred to as a “seesaw effect” [6], may help explain the enhancement of recognition memory observed here. Of particular relevance, animal lesion studies have revealed that damage to one system can actually *enhance* learning by the other [63], [64], [65]. Such findings have been taken as evidence for a competitive relationship between the two systems that may interfere with learning and processing under normal conditions. The enhanced performance of the unimpaired system has been proposed to reflect the removal of interference by the damaged system [63]. Neuroimaging studies suggest the existence of similar competitive mechanisms in humans [59], [66], [67]. Since it has been proposed that procedural memory is impaired in DD [6], [11], a prediction that is supported by a number of studies [11], [12], [14], [15], [68], [69], [70], [71], [72], it is possible that the declarative memory enhancement found in the present study may be partially explained by such competitive interactions.

A possible enhancement of aspects of declarative memory is particularly intriguing since evidence suggests that declarative memory may play an important compensatory role for the reading deficits in dyslexia. This phenomenon may reflect the cooperative aspect of the procedural-declarative relationship [6], [61], [62], [73], [74]. For example, previous studies indicate that persistent phonological decoding problems in individuals with DD may be associated with an increased reliance on chunking and whole word memorization for reading [75], [76]. In addition, behavioral interventions have been found to lead not only to reading improvements, but also to changes in the hippocampus and other medial temporal lobe structures, in both functional and structural imaging studies [77], [78]. Similar compensatory mechanisms have previously been reported for populations afflicted with procedural memory impairments (e.g. Parkinson’s disease) in which declarative memory has been shown to take over certain cognitive functions that are normally performed by the procedural memory system [73], [74], [79], [80], [81].

If declarative memory indeed plays a compensatory role for reading in the present study, we would expect to find a correlation between performance at the recognition memory test and reading scores in the DD group, but not in the TD group. This prediction did not seem to be strictly supported, as the correlation between recognition memory d’ scores (across both sessions) and the combined standardized raw scores from the two reading tests did not reach significance. However, the lack of such an effect in the DD group may reflect a lack of power due to the small sample size. Indeed, the correlation approached significance in the DD group (*p* = .061) but not in the TD group (*p* = .774). Moreover, the difference between the two correlations was borderline significant (*p* = .056). Thus, the investigation of a potentially compensatory role of declarative memory in DD requires further studies with larger samples.

The present study has various limitations that may be addressed by future studies. First, samples sizes were relatively small. Thus, the present findings need to be replicated with larger samples in order to ensure their generalizability. In addition, the present study tests only one aspect of declarative memory, and leaves many other aspects unexplored. Future studies would benefit from contrasting the recognition memory paradigm used in the present study with paradigms assessing encoding strategies and recall. Such a within-subjects design would allow for a powerful examination of different aspects of declarative memory, and their status in DD.

Keeping these limitations in mind, the results of the present study are encouraging, as they point to possible cognitive strengths associated with an otherwise difficult and persisting condition. Crucially, knowledge about which aspects of cognition are intact, or even enhanced, in DD, should be of theoretical as well as of clinical and pedagogical interest.
