# Correction: Enhanced Recognition Memory after Incidental Encoding in Children with Developmental Dyslexia

**DOI:** 10.1371/annotation/925d6161-da3b-46f5-9946-e3cfec7a1681

**Published:** 2013-12-16

**Authors:** Martina Hedenius, Michael T. Ullman, Per A. Alm, Margareta Jennische, Jonas Persson

There are several errors in the author information.

The Corresponding Author should be Martina Hedenius. The email address, martina.hedenius@neuro.uu.se, is correct.

The third author's name is spelled incorrectly. The correct name is Per A. Alm.

Three authors, Michael T. Ullman, Margareta Jennische and Jonas Persson, should be noted as joint senior authors of this work.

